# Syndrome de Heerfordt: à propos d’une observation et revue de la littérature

**DOI:** 10.11604/pamj.2020.37.117.25338

**Published:** 2020-10-02

**Authors:** Smail Kharoubi

**Affiliations:** 1 Service ORL, CHU Annaba Faculté de Médecine, Universite Badji Mokhtar, Annaba, Algérie

**Keywords:** Granulomatose, parotidomégalie, paralysie faciale, enzyme de conversion, angiotensine, Granulomatosis, parotidomegaly, facial paralysis, converting enzyme, angiotensin

## Abstract

Le syndrome de Heerfordt est une manifestation rare de la sarcoïdose associant dans sa forme classique une uvéite, parotidomégalie, paralysie faciale et fièvre. C'est une forme active de la maladie dont le diagnostic est facilité par la biopsie salivaire. Il s'agit d'une observation clinique d'une patiente âgée de 17 ans présentant une uvéite, une parotidomégalie droite et une paralysie faciale droite d'apparition brutale. Après un bilan biologique et une imagerie (échographie parotidienne), une biopsie des glandes salivaires accessoires a conduit au diagnostic de sarcoïdose. Un bilan pré thérapeutique a permis d'instituer une corticothérapie par voie orale avec une évolution favorable et rémission totale. Le syndrome de Heerfordt est une forme clinique rare de sarcoïdose avec un profil évolutif favorable dans la majorité des cas. Il nécessite une approche diagnostique précise (éliminer les diagnostics différentiels surtout les formes incomplètes) en insistant sur les progrès thérapeutiques dans ce domaine.

## Abstract (en)

Heerfordt's syndrome is a rare manifestation of sarcoidosis combining uveitis, parotidomegaly, facial paralysis and fever in its classic form. It is an active form of the disease whose diagnosis is facilitated by salivary gland biopsy. We conducted clinical observation of a 17-year-old female patient with uveitis, right parotidomegaly and right facial paralysis characterized by violent onset. After laboratory tests and imaging exam (parotid ultrasound), biopsy of minor salivary glands established the diagnosis of sarcoidosis. A pre-therapeutic assessment allowed for the initiation of oral corticosteroid therapy with favorable outcome and total remission. Heerfordt's syndrome is a rare clinical form of sarcoidosis, with favorable outcome in most cases. Specific diagnostic approach (excluding differential diagnoses, especially the incomplete forms) is necessary, based on therapeutic advances in this area.

## Introduction

La sarcoïdose est une maladie inflammatoire chronique avec une incidence de 3 à 10 pour 100 000. Elle est multifocale mais reste dominée par ses manifestations respiratoires (médiastino pulmonaires). En 1909, Heerfordt avait décrit une entité syndromique associant uvéite, parotidite et paralysie faciale dans un contexte fébrile [1]. Waldenstrom en 1937 avait rattaché ce syndrome à la sarcoïdose [2]. C´est une manifestation rare de la maladie de diagnostic facile dans sa forme complète et repose sur l´étude histopathologique d´un prélèvement salivaire. Le traitement repose sur la corticothérapie avec une évolution très souvent favorable.

## Patient et observation

**Figure 1:**
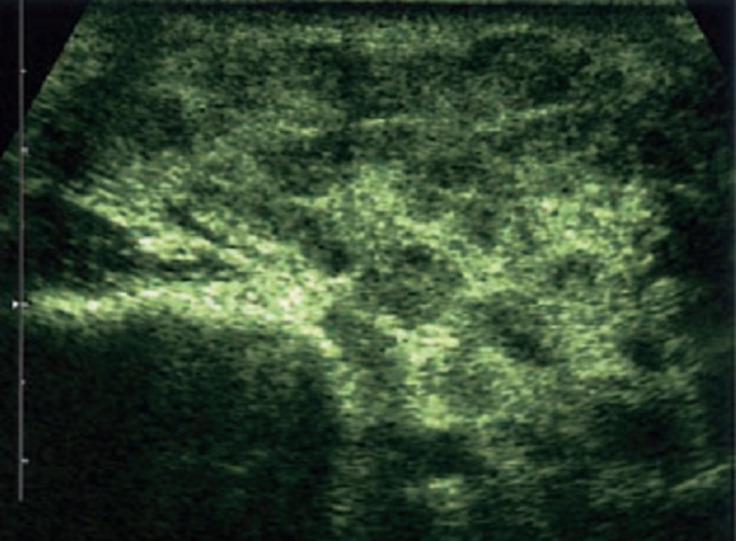
échographie parotidienne; hypertrophie diffuse hypo échogène avec cloisonnements (septa)

**Figure 2:**
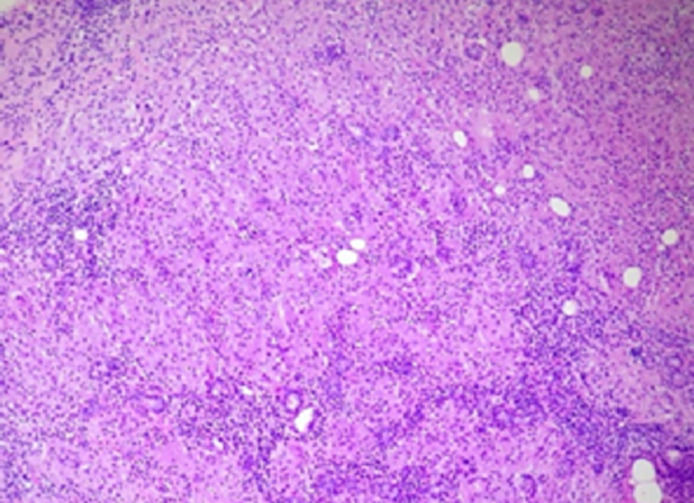
granulome sans nécrose formé par la confluence de nodules comportant des histiocytes, des cellules géantes à corps étranger avec des lymphocytes et canalicules salivaires

SA âgée de 17 ans hospitalisée en ophtalmologie pour une uvéite est adressée en ORL pour une asymétrie faciale évoluant depuis 4 jours. Patiente correctement vaccinée sans antécédents particuliers, avait présenté une rougeur oculaire avec des troubles visuels (œil droit) depuis une semaine. L´asymétrie faciale droite était d´apparition brutale. L´état général était conservé avec une température à 37,5°C. L´examen de la face avait montré au repos une asymétrie faciale droite aggravée lors des mouvements avec un signe de Charles-Bell positif. La paralysie faciale était cotée à VI (House-Brackman). L´examen avait noté également une tuméfaction diffuse et régulière de la parotide droite, indolore sans modifications cutanées avec un orifice buccal du canal de sténon propre. Le bilan biologique avait montré une vitesse de sédimentation (VS) à 25/32mm; protéine C réactive (CRP): 10mg/l; globules blancs à 6 x 109/l; globules rouges 3 millions par microl; hémoglobine 13,4g/dl, urée et créatinine normales. Albuminémie à 4,5g/dl et un taux de gamma globulines de 18g/l. L´échographie cervicale avait montré une hypertrophie diffuse hypoéchogéne de la glande parotide droite avec quelques ganglions infracentimétriques. Le telethorax était normal (Figure 1). Devant cette triade clinique (uvéite, paralysie faciale et parotidite) un syndrome de Heerfordt était évoqué et une biopsie des glandes salivaires accessoires (labiales) était réalisée. L´examen anatomopathologique avait noté la présence d´une réaction inflammatoire avec granulome giganto cellulaire sans nécrose caséeuse (Figure 2). Un bilan pré thérapeutique était réalisé: scanner thoracique sans anomalies (médiastin), l'intradermo-réaction (IDR) à la tuberculine négative, bilan phospho calcique normal, dosage de l´angiotensine était de 32U/l (30 à 100U/l). Une corticothérapie 1mg/kg de prednisolone était institué avec des mesures de protection oculaires. Un contrôle clinique à 2 mois avait constaté une régression totale de la paralysie faciale et de la parotidite. L´évolution était satisfaisante après 13 mois de recul.

## Discussion

Le syndrome de Heerfordt est une entité rare et représente 5 à 10% des sarcoïdoses et 4,4 à 5,6% pour Fukuhara *et al*. [3]. Il est considéré comme une forme active et extensive de la sarcoïdose. On définit classiquement la forme complète ou les quatre symptômes sont présents (uvéite, parotidomégalie, paralysie faciale, fièvre) et les formes incomplètes avec deux ou trois critères. Il intéresse l´adulte, âge moyen 40 ans, de sexe féminin surtout d´origine scandinave. Entre 2003 et 2013, 31 articles faisant référence au syndrome de Heerfordt ont été rapportés [4]. Notre revue de la littérature a porté ce chiffre à 42 publications. Sur le plan pathogénique, les lésions sont d´origine auto immune en réaction à un antigène inconnu (bactérie, virus) conduisant à un désordre cytokinique, en particulier une augmentation de la sécrétion de *Tumor Necrosis Factor* (TNFα), qui participe à la formation de granulomes dans différents organes. Habituellement sporadique, SNELL avait décrit deux observations de syndrome de Heerfordt chez deux sœurs [5]. Génétiquement, l'allèle HLA-DRB1*04 semble être protecteur contre la sarcoïdose et facilitateur par rapport à la survenue du syndrome de Heerfordt [6]. Le tableau clinique associe dans sa forme complète une uvéite, une parotidite bilatérale récidivante et une paralysie faciale périphérique dans un contexte fébrile ou subfébrile. Les manifestations ophtalmologiques sont constantes, inaugurales sous un mode aigu associant une photophobie, un larmoiement et une sécheresse oculaire. Il s´agit surtout d´une uvéite antérieure assez caractéristique. D´autres lésions ont été décrites: nodules conjonctivaux, uvéite intermédiaire (vitré et pars planite) voire une uvéite postérieure et choriorétinite.

L´atteinte parotidienne se voit dans 65% des cas sous forme d´une tuméfaction diffuse récidivante et indolore avec souvent une xérostomie. Elle est parfois bilatérale et peut atteindre les glandes sous mandibulaires et lacrymales [3]. La paralysie faciale est inconstante et se voit dans 25 à 50% des cas. Elle est secondaire à l´atteinte de l´épinerve par les granulomes sarcoïdosiques et à l´inflammation périneurale. Des mécanismes vasculaires (ischémiques) voire l´atteinte des branches intra parotidiennes du nerf facial ont été rapportées [3,4]. Sa survenue est brutale, de type périphérique, apparait après la tuméfaction parotidienne et peut être à bascule ou bilatérale dans 0,3 à 2% [7]. A côté de ces formes typiques, il y a plusieurs observations de formes incomplètes comportant un ou deux éléments de la triade. Sur le plan général, une asthénie est notée dans 50 à 70% des cas avec une fièvre variable entre 38 et 38,5°C pouvant évoluer sur plusieurs jours. Pour certains auteurs c´est un élément diagnostic important du syndrome de Heerfordt [4]. Sur le plan biologique la VS et CRP peuvent être normales ou légèrement élevées. La formule numération sanguine montre souvent une lymphopénie (lymphocytes T) avec souvent un rapport CD4+/CD8+ sanguin diminué. On note une hyper gamma globulinémie polyclonale dans 30 à 80% des cas. La présence d'auto anticorps peut se voir chez certains patients (ANCA, ANA). Le métabolisme phosphocalcique est souvent perturbé; hypercalcémie modérée avec surtout une hypercalciurie.

Il est classique de noter une élévation de l´enzyme de conversion de l´angiotensine (ECA) dans 60% des cas. Cette élévation est liée à une production anormale d'ECA par les macrophages activés et les cellules épithélioïdes dans les granulomes. La réduction du taux d´ECA sous corticoïdes confirme le diagnostic sans pour autant constituer un critère pronostic. D´autres biomarqueurs sont corrélés à l´activité sarcoïdosique: lysosyme, IL2R sérique, B2microglobuline, chitotriosidase, KL-6 [3,4]. Le dosage des IgG4 est intéressant pour isoler les maladies à IgG4 (IgG4-related disease) qui peuvent s´associer à une atteinte des glandes salivaires (sialites), lacrymales voire une paralysie faciale. Ces atteintes peuvent mimer en tout point un syndrome de Heerfordt qui s´accompagne habituellement d´un taux normal d´IgG4 (0,08 a1, 40 g/l) [8]. Le *Tumor Necrosis Factor* (TNF) alpha est un bon marqueur de l'activité inflammatoire granulomateuse et son taux est très souvent élevé (normal moins de 2,8pg/ml). C'est un bon indice de l'évolutivité de la maladie, du suivi thérapeutique (normalisation) voir pathogénique dans certaines formes réfractaires aux corticoïdes et qui répondent favorablement aux anti-TNF [9]. L´IDR à la tuberculine est négative dans 80% des cas (anergie tuberculeuse) et prend toute sa valeur en cas de positivité antérieure. Le Quantiféron est également négatif. L´imagerie comporte l´exploration des glandes salivaires (échographie et scanner) montrant une hypertrophie diffuse, régulière souvent cloisonnée par des septa et une hypervascularisation en mode doppler [10].

L'imagerie par résonance magnétique (IRM) montre un hyper signal parotidien après injection de gadolinium avec hypertrophie des glandes lacrymales. Le scanner thoracique recherchera une atteinte médiastino-pulmonaire et permet une stadification de la maladie. La scintigraphie au [^18^F]-fluorodésoxyglucose montre une hyperfixation au niveau des foyers sarcoïdosiques actifs et permet si nécessaire d'orienter les biopsies [10]. La cytologie parotidienne peut aider au diagnostic surtout en mode écho guidé qui facilite également la réalisation des micro biopsies [10]. Le diagnostic repose sur la biopsie des glandes salivaires; parotide (à risque) mais surtout accessoires spécifiques dans 60% des cas. Elle montre un granulome giganto cellulaire sans nécrose caséeuse. Le granulome est composé d'une couronne lymphocytaire entourant un follicule central constitué essentiellement de cellules épithélioïdes associées à des cellules géantes et quelques lymphocytes. La majorité des lymphocytes situés dans la partie centrale des granulomes sont des lymphocytes CD4 mais la périphérie des granulomes est composée de lymphocytes CD4 et de lymphocytes CD8. Plusieurs associations syndromiques ont été décrites dans la littérature: syndrome de Heerfordt - syndrome de Gougerot-Sjogren, atteinte du nerf trijumeau, paralysie du VI, péricardite, leukoencéphalopathie multiple progressive, otalgies, adénopathies pré auriculaires, atteinte des ganglions de Gasser, œdème bilatéral des paupières, atteinte cutanée, hyperacousie, vertige, dysgueusie, dysosmie [11-15].

Sur le plan évolutif la régression spontanée est possible. Le traitement permet une rémission des signes cliniques sur une période allant de 12 à 36 mois. La survenue d´une localisation pulmonaire et la diffusion de la maladie peuvent se voir à long terme. La mortalité est entre 1 à 5% [16]. Le diagnostic différentiel se pose avec une tuberculose, la maladie de Wegener; les lymphomes malins, la maladie de Castleman. La prise en charge thérapeutique est essentiellement médicale et trouve toute son indication d´emblée devant une atteinte oculaire et neurologique (paralysie faciale). La corticothérapie représente le traitement de première ligne du syndrome de Heerfordt. La voie systémique avec une dose de 20 à 40 mg/jour (certains cas peuvent bénéficier de 80 mg/j) pendant 1 à 3 mois puis diminuer la dose de 5 à 10 mg/j toutes les 2 à 4 semaines pour atteindre une dose d'entretien de 5 à 10 mg/jour pendant 6 à 9 mois [17]. En cas de progression de la maladie ou d'effets secondaires majeurs on bascule vers un protocole de seconde ligne. Les anti métabolites constituent le traitement de seconde ligne: azathioprine, méthotrexate.

Leur mécanisme d´action consiste à freiner la prolifération lymphocytaire. A titre indicatif le méthotrexate est utilisé à la dose de 5 à 15 mg/semaine per os avec une augmentation de 5 mg/semaine tous les 3 mois (sans dépasser 20 mg/semaine) pendant une durée de 6 mois. On y associe 5 mg/semaine d'acide folique pour contrôler les effets secondaires [18]. L'azathioprine est prescrite à la dose de 150mg/j (50-200mg/j). D´autres molécules peuvent également être utilisées en seconde ligne de traitement avec un impact plus faible: la chloroquine, l´hydroxychloroquine 200 à 400 mg/j, le mycophénolatemofétil 2000mg/j ou le léflunomide 10 à 20 mg/j [17]. Les traitements dits “biologiques” constituent la troisième ligne. Ceux sont les anti-TNF alpha représentés essentiellement par l'infliximab [16]. Un taux de rechutes important a été noté à l'arrêt du traitement. L´adalimumab et le golimumab sont des anticorps monoclonaux humains recombinant qui agissent selon le même mécanisme que l´infliximab. Dans tous les cas le sevrage de ces molécules doit être progressif et s'étaler sur deux ans après son introduction. Sur le plan de la stratégie de prise en charge il faut respecter l'escalade hiérarchisée des trois niveaux de traitement avec un suivi prolongé clinique et biologique en raison de l´incertitude évolutive (survenue de formes réfractaires, graves) et de la morbidité potentielle de ces protocoles.

## Conclusion

Le syndrome de Heerfordt est une forme rare de sarcoïdose. Le diagnostic est simple dans sa forme complète et repose sur la biopsie des glandes salivaires. L´évolution est globalement favorable sous traitement médical avec peu de séquelles fonctionnelles (oculaires, nerf facial). De nouvelles acquisitions génétiques et biologiques ont permis de clarifier et de mieux encadrer cette pathologie (facteurs pronostics et évolutifs). Les formes réfractaires, diffuses ou associées à d´autres affections auto immunes nécessitent une approche adaptée et multidisciplinaire.

## References

[1] Heerfordt CF (1909). "Übereine, “Febrisuveo-parotide asubchronica” an der Glandulaparotisund der Uvea des Auges lokalisiertundhaufug mit Paresencerebrospinaler Nervenkompliziert. Albrecht von Grafes Archivfür Ophthalmologie.

[2] Waldenstrom JG (1937). Some observations of uveoparotitis and allied conditions with special reference to the symptoms from the nervous system. Acta Med Scand.

[3] Fukuhara K, Fukuhara A, Tsugawa J, Oma S, Tsuboi Y (2013). Radiculopathy in patients with Heerfordt´s syndrome: two case presentations and review of the literature. Brain Nerve.

[4] Chappity P, Kumar R, Sahoo AK (2015). Heerfordt's syndrome presenting with recurrent facial nerve palsy: case report and 10-year literature review. Sultan QaboosUniv Med J.

[5] Snell NI, Karlish A (1975). Heerfordt's syndrome in two sisters. Br Med J.

[6] Darlington P, Tallstedt L, Padyukov L, Kockum I, Cederlund K, Eklund A (2011). HLA-DRB1* alleles and symptoms associated with Heerfordt´s syndrome in sarcoidosis. Eur Respir J.

[7] Srirangaramasamy J, Kathirvelu S (2019). A rare case of heerfordt's syndrome with bilateral facial palsy. Indian J Otolaryngol Head Neck Surg.

[8] Puxeddu I, Capecchi R, Carta F, Tavoni AG, Migliorini P, Puxeddu R (2018). Salivary gland pathology in IgG4-related disease: a comprehensive review. J Immunol Res.

[9] Makimoto G, Miyahara N, Yoshikawa M, Taniguchi A, Kanehiro A, Tanimoto M (2016). Heerfordt's syndrome associated with a high fever and elevation of TNF-α. Acta Med Okayama.

[10] Fischer T, Filimonow S, Petersein J, Zimmer C, Beyersdorff D, Guski H (2002). Diagnosis of Heerfordt's syndrome by state-of-the-art ultrasound in combination with parotid biopsy: a case report. Eur Radiol.

[11] Blair MP, Rizen M (2005). Heerfordt syndrome with internal ophthalmoplegia. Arch Ophthalmol.

[12] Otani K, Noda K, Ukichi T, Kingetsu I, Kurosaka D (2013). A case of abortive type of Heerfordt syndrome associated with paralysis of trigeminal nerve. Nihon Rinsho Meneki Gakkai Kaishi.

[13] Shiraishi K, Sadamoto Y, Sayama K (2019). Heerfordt syndrome developing in a patient with cutaneous sarcoidosis. Australas J Dermatol.

[14] Yagi T, Hattori H, Ohira M, Nakamichi K, Takayama-Ito M, Saijo M (2010). Progressive multifocal leukoencephalopathy developed in incomplete Heerfordt syndrome, a rare manifestation of sarcoidosis, without steroid therapy responding to cidofovir. Clin Neurol Neurosurg.

[15] Fujita H, Hiraide F, Kawano A (1997). A case of incomplete Heerfordt syndrome accompanied by dysosmia. Practica Oto-Rhino-Laryngologica.

[16] Daldon PEC, Arruda LHF (2007). Granulomas nao-infecciosos: sarcoidose. An Bras Dermatol.

[17] Nunes H, Pigne E, Soler P, Valeyre D (2003). Traitement de la sarcoïdose. La Lettre du Pneumologue.

[18] Cremers JP, Drent M, Bast A, Shigemitsu H, Baughman RP, Valeyre D (2013). Multinational evidenced-based World Association of Sarcoidosis and Other Granulomatous disorders recommendations for the use of methotrexate in sarcoidosis: Integrating systematic literature research and expert opinion of sarcoidologists worldwide. Curr Opin Pulm Med.

